# Natural Killer Cell Development and Maturation Revisited: Possible Implications of a Novel Distinct Lin^−^CD34^+^DNAM-1^bright^CXCR4^+^ Cell Progenitor

**DOI:** 10.3389/fimmu.2017.00268

**Published:** 2017-03-09

**Authors:** Federica Bozzano, Francesco Marras, Andrea De Maria

**Affiliations:** ^1^Department of Experimental Medicine (DIMES), University of Genova, Genova, Italy; ^2^Center of Excellence for Biomedical Research (CEBR), University of Genova, Genova, Italy; ^3^Istituto Giannina Gaslini, Genova, Italy; ^4^Clinica Malattie Infettive, IRCCS AOU San Martino-IST Genova, Istituto Nazionale per la Ricerca sul Cancro, Genova, Italy; ^5^Department of Health Sciences, DISSAL, University of Genova, Genova, Italy

**Keywords:** natural killer, NK cell development, CD34, DNAM-1, common lymphoid progenitors

## Abstract

Since the first description of natural killer (NK) cells, the view on their role in innate immunity has evolved considerably. In addition to first-line defense against transformed and pathogen-infected autologous cells, NK cells contribute to modulate adaptive immune responses and in some cases acquire specialized functions, including exhausted, adaptive, and decidual NK cells. NK cells derive from CD34^+^ progenitors, *in vivo* and *in vitro*; however, it is unclear whether the high phenotype diversity *in vivo* may be generated from these precursors alone. The recent characterization of a novel CD34^+^DNAM-1^bright^CXCR4^+^ precursor giving rise to apparently licensed and functional maturing NK cells may suggest the possibility for a higher than expected common lymphocyte precursor diversity and a consequently higher peripheral NK cell phenotype variability. Here, we review the evidences on NK cell central and peripheral development from CD34^+^ precursors and propose a possible updated reading frame based on the characterization of CD34^+^DNAM-1^bright^CXCR4^+^ cell progenies, which favors the possibility of concurrent NK cell maturation from different CD34^+^ precursors.

## Introduction

Natural killer (NK) cells, are a central component of the innate immune response (1) and constitute the first line of defense against a variety of tumors and microbial pathogens (2–5).

Over recent years, it has become clear that their role exceeds the boundaries of the original assignment to patrol the tissues as a first line of defense and rather also includes regulatory and editing functions of the innate and adaptive immune response.

Opposite to T or B cells, NK cells do not undergo somatic rearrangement of genes coding for antigen-specific receptors. Their functional characteristics include production and release of IFNγ and TNFα and also G-CSF. In addition they may produce chemokines and IL-8, and under specialized and limited conditions also IL-10, IL-6, and IL-1. Following the identification and characterization of innate lymphoid cells (ILCs) into at least three lineages characterized by different phenotype, homing, and function (6, 7), NK cells have been proposed to be included into group 1 ILC (1) based on their expression of NFIL3, Tbet, and Eomes transcription factors (1, 8).

Human NK cells normally constitute 5–15% of peripheral blood (PB) lymphocytes. The majority of NK cells, are present in relative abundance in bone marrow (BM), liver, uterus, spleen, and lungs, as well as to a lesser extent in secondary lymphoid tissue (SLT), mucosa-associated lymphoid tissue, and the thymus. The classical description of NK cell phenotypes relies on CD56 and CD16 expression with the distinction of three broad phenotypes which include CD16^+/−^CD56^bright^ cells representing a minority of circulating and the majority of tissue-associated NK cells, CD16^+^CD56^dim^ cells that constitute the majority of circulating NK cells and are viewed as effectors cells, and CD16^+^CD56^−^ exhausted NK cells that are poorly functional express low levels of natural cytotoxicity receptors (NCRs) and may become more abundant in PB during chronic infections, such as HIV-1 infection. A peripheral development of NK cells has been shown to take place beyond BM and lymphnode niches. NK cells undergo a progressive development of CD56^bright^ into CD56^dim^ NK cells with a progressive loss of NKG2A and concomitant progressive expression of KIRs, CD57, and NKG2C into terminally differentiated NK cells (9–11).

The original view encompassing a tripartite subset characterization has recently been updated in view of NK cell phenotype diversity. Using mass cytometry, it has become evident that in healthy adult humans a much larger number of distinct NK cell phenotypes are simultaneously present at any time. From 6,000 to 30,000 different NK cell phenotypes have been identified by mass in a single donor, and in any small group of persons up to 100,000 different NK cell phenotypes may be detected (12). In addition, by computer-assisted flow-cytometric analysis, at least 5–8 distinct subsets may be identified using a set of three NK cells specific monoclonal antibodies beyond CD16 and CD56 (11). This abundance of NK cell phenotype diversity is determined by combinatorial expression of the multitude of receptors and co-receptors present on their surface. In this regard, there are both evidences for an inherent intrinsic or genetically determined driver for the persistence of some phenotypes (mostly accounting for KIR variability), and for an extrinsic or environmental influence on the prevalence of other phenotypes supported by foreign antigenic stimulation supporting the diversity for NCR representation and expression (12).

## CD34 NK Cell Precursors

Similar to other blood cells, NK cells derive from hematopoietic stem cells (HSCs) and can be grown *in vitro* from lymphoid-restricted multipotent progenitors that may retain B and/or T lymphocyte developmental potential (13–15). The classical model of hematopoiesis postulates that the earliest fate decision toward NK cells downstream of HSCs is represented by the divergence of lymphoid and myeloid lineages. Erythroid and megakaryocyte lineages branch off before the lymphoid–myeloid split. This step is followed by myeloid–lymphoid divergence where common lymphoid progenitors (CLPs), and common myeloid progenitors (6) are generated. Accordingly, the CLP group would not include cell progenitors with myeloid potential. In contrast to mouse hematopoiesis, definitive evidence for a comprehensive model that best describes human hematopoiesis is still to be completely defined (16). Recently, a different pattern of cell maturation has been proposed following *ex vivo* and *in vivo* results in humans. Analysis of human cord blood (CB) and BM using seven distinct markers, including CD45RA, CD135 (Flt3), CD7, CD10, CD38, and CD90, allowed the identification of seven distinct progenitor cell classes (17). In this setting, some cells are described as multi-lymphoid progenitors (MLPs), defined by CD34^+^CD38^−^Thy-1^neg–low^CD45RA^+^, belong to the CLP group and are able, in specific culture conditions, to give rise to all lymphoid cells as well as monocytes, macrophages, and dendritic cells (DCs) (18, 19). Among these MLPs included in this last model, NK cells derive from CD34^+^ hematopoietic stem cells (HPC) precursors originally identified in BM (20). However, CD34^+^ cells giving rise to NK cell progeny have been detected also in PB, thymus, lymphnodes, CB, GALT, and decidua (21, 22). In addition, other reports indicate that T and NK cells are generated from non-characterized bipotent T/NK common progenitors, which may circulate in PB of healthy donors (HDs), albeit at very low frequencies (23, 24). While it is agreed that CD34^+^ NK cell progenitors reside in the BM, there is a less clear view on whether seeding of these cells into other organs generates organ-specific NK cell maturation, or whether a predefined CLP or MLP with specific developmental and homing characteristics would exit under certain conditions from the BM and specifically seed into the final sites of maturation.

## NK Cell Maturation

Distinct stages of development of NK cells from HPC have been described with an orderly and staged acquisition of NK cell markers, and distinct maturational stages (1). Five stages of human NK cell development have been described (25). Stage 1–2 CD34^+^CD45RA^−/+^Cd10^+/−^CD117^−/+^ cells have been observed in human SLT and retain non-NK cell lineage potential since under optimal *in vitro* conditions they can develop into T and DC cells. This development potential is lost in the third stage in which may identify committed immature NK (iNK) cells.

The acquisition of the interleukin 15 (IL-15R) receptor beta chain (CD122) marks an important step of NK cell differentiation, since IL15 promotes NK cell differentiation, functional maturation, and survival in both mouse and human (26). Thus, IL-15R expression identifies an NK cell precursor subset defined by developmental potential in response to IL-15, by lack of functional immunophenotype observed in mature NK cells and by lack of other Lineage specific surface antigen as CD3, CD14, and CD19. Two populations of IL-15-responsive Lin^−^CD94^−^NK differentiating intermediates have been identified (Lin^−^CD34^dim^CD45RA^+^ alpha4beta7^bright^CD117^+^CD161^+/−^CD94^−^ stage 2 and Lin^−^CD34^−^ alpha4beta7^−^CD117^+^CD161^+^CD94^−^ stage 3). They are enriched in the interfollicular T cell-rich areas of secondary lymphoid organs where their putative progeny, CD56^bright^CD94^+^ NK cells, also resides (25, 27, 28). This anatomical localization has been attributed to specific trafficking of BM derived NK cell precursors to SLT *via* high endothelial venules and would be mediated by high expression of CD62L on circulating Lin^−^CD94^−^NK differentiating intermediates (28). NK cell differentiation then progresses by orderly acquisition of CD161, CD56, CD94/NKG2A, NKp46, NKG2D, KIRs and functional receptors CD16 (27, 29, 30). The role of CD56 during NK cell development is yet undefined while acquisition of CD94, which then persists on PB CD56^bright^ NK cells and is needed for surface expression of NKG2A or NKG2C, signals the transition to stage 4 in SLT and NK cell maturation is completed with transition from stage 4 CD56^bright^CD16^+/−^ to stage 5 in CD56^dim^CD16^+^ NK cells (9, 31). A source of possible confusion is represented by work showing that cells of myeloid lineage may, under certain specific conditions, generate NK cells *in vitro* (32). This work concentrates only on cord-blood CD34^+^ cells under particular conditions (32). The view that NK cells may be derived *in vitro* together with myelomonocytes without evidence for T cell growth reflects work by other groups as well (27, 33).

Some caution is needed when considering these models, which are nevertheless useful to provide a general scaffolding to understand NK cell peripheral maturation. Opposite to the model of peripheral T cell maturation from which it has been shifted for practical purposes, NK cell maturative migration between stages is not a one-way process. For example, NK cells may revert form terminal differentiation and, under favorable conditions *in vitro* (e.g., IL18 supplementation), may modify surface receptor expression with upregulation of CCR7, CD83, and CD25 and downregulation of CD16 (34). Furthermore, *in vivo*, NK cell education, epistatic interaction with KIR genes and viral infection or other environmental stimuli have a marked bearing on NK cell repertoire phenotype and activating and inhibitory receptor expression (35, 36).

An additional layer of entanglement to a linear model of NK cell development has been represented by the suggestion that NK cells may represent a subset of ILC (37). ILC have been shown *in vitro* derived from CD34^+^ cells isolated preferentially from the CB compared to PB (38). Indeed, the recent demonstration of the possibility of an elective ILC deficiency in humans without NK cell deficiency shows that ILCs might be dispensable in natural conditions and that developmental pathways for NK cell and ILC development are distinct (37).

## Outliers to a Linear Single-Cell Model of NK Cell Development

There are additional outliers to a model of sequential NK cell development that cannot be apparently reconciled with a single-cell maturation scheme for NK cells, so far. These include the observation of adaptive or memory-like NK cell responses, the appearance of CD56^−^CD16^+^ exhausted NK cells in some clinical conditions, and the origin of NK cells in decidua.

In mice, infection with MCMV determines the expansion of specific NK cell subsets (39–41), which maintain for prolonged periods of time the ability to produce increased amounts of TNFα and IFNγ. This observation is reminiscent of memory T cell function thus suggesting a possible memory-like or adaptive feature of NK cells. This pattern has been observed also in humans and predominantly relates to HCMV previous infection (42–45). Human adaptive NK cell expansions are monomorphic. Indeed, only increased proportions of NKG2C^+^ cells appear in PB, irrespective of the different invading pathogens that have been so far able to induce such NK cell expansions, including HCMV or Hantavirus or Chikungunya (46, 47). NKG2C^+^ NK cells expansions persist (48) after acute HCMV infection into latency, and may be observed also after BM transplantation (48, 49). Similar to virus-induced adaptive NK cells (50, 51), NKG2C^+^ NK cells may be obtained after cytokine induction (52). Active research in this area so far did not reach conclusive evidence that these memory-like NK cell expansions occur as a single terminal event along the previously described pathway of peripheral NK cell development. Additional work and efforts are needed to directly answer some crucial questions in this area. Specific trials and work will need to be designed to understand (a) whether the increase in adaptiveNKG2C^+^ NK cells during Hantavirus or other RNA virus infections represents an HCMV-independent event, or rather reflects a recall response of NKG2C^+^ adaptive NK cells in HCMV^+^ patients with latent infection, (b) whether only viruses or rather other pathogens may associate with NKG2C^+^ NK cell expansions, (c) why only NKG2C^+^ adaptive NK cells represent a recall response to invading viruses with different antigenic and PAMP characteristics (e.g., HCMV is a DNA-virus, Chikungunya is a RNA virus, no shared molecular patterns have been described), and (d) what is the advantage in terms of virus control or host survival provided by this quite specific HCMV-associated adaptive NK cell response.

Another apparent outlier to sequential NK cell subset development is represented by CD56^neg^CD16^+^ exhausted NK cells, which have been described for the first time over 20 years ago (53). These cells may represent up to 20–40% of all NK cells or 3–6% of all lymphocytes in HIV^+^ patients while they represent a rare population in the PB of HDs. Immunophenotypic analyses revealed that cell surface receptors expressed on CD56^neg^CD16^+^ cells overlap with that of so-called “stage 3” iNK cells and are able, albeit to a reduced extent, to kill target cells and produce chemokines (54). Thus, it appears unlikely that CD56^neg^ NK cells represent the progeny of iNK cells. Effector molecule expression by CD56^neg^ NK cells further support the possibility that these cells are more closely related to and share characteristics with more highly differentiated CD56^dim^ NK cells. Additional comprehensive studies of this subset are needed, in order to clarify when and under which stimuli aberrant differentiation into CD56^neg^CD16^+^ NK cells occurs as well as whether it is reversible or not.

Natural killer cells also localize in differentiated tissues including non-pregnant endometrium (55). Following embryo implantation, decidualization of human endometrium is associated with a massive recruitment of NK cells that will build up and may represent as many as 50–90% of lymphoid cells present in decidual tissue. Decidual NK cells (dNK) numbers progressively decrease from mid-gestation onwards (56). dNK cells have unique phenotypic properties and functional profile and are CD56^bright^CD16^+/−^ KIR^+^ cells.

Analysis of gene expression in dNK revealed relevant differences with both CD56^bright^ and CD56^dim^ peripheral NK cell subset. CD9, galectin, alpha-1 integrin, and other adhesion molecules are over-expressed in dNK (57), express major activating NK receptors, including NKp46, NKp30, NKG2D, and DNAM-1, and contain high levels of perforin and granzymes (comparable to CD56^dim^ peripheral NK cells), but have a poor ability to kill classical NK target cells (22, 58–60). dNK cells are able to release high amounts of cytokines and chemokines (including IL-8, VEGF, SDF-1, and IP-10), which are involved in tissue remodeling, trophoblast migration, and/or neo-angiogenesis and placentation. Thus, dNK cells appear to play an unexpected role in vessel formation and tissue building rather than their classical defensive role and these findings further underline the plasticity of NK cells, possibly induced by the particular microenvironment. Whether dNK cells represent a peripheral differentiation of other NK subsets or whether they represent a specific lineage derivation from a common precursor is so far poorly understood.

## A Novel CD34^+^ Precursor and a New Perspective

Recently, a novel, previously uncharacterized CLP has been identified and is defined by Lin^−^CD34^+^ DNAM-1^bright^CXCR4^+^ markers (60). These precursors were found enriched in PB of patients with chronic inflammatory conditions of either infectious or non-infectious origin. Based on available data on CD34^+^ cell maturation niches (61–64), the phenotype of these cells suggest that they represent recent migrants from BM that still retain CXCR4 and DNAM-1, which are derived from endosteal niches following bone remodeling during chronic inflammation (65, 66). In HSC, BM donor Lin^−^CD34^+^DNAM-1^bright^ cells represent only a fraction (15%) of mobilized CD34^+^ cells. A comparison of chemokine receptor expression provided clues to a different end-organ circulation of these cells compared to classical CD34^+^DNAM^−^ progenitors. Lin^−^CD34^+^DNAM-1^bright^CXCR4^+^ cells appear to have the potential of trafficking not only into lymphnodes or GALT *via* CD62L/L interactions, but also (or rather) to peripheral inflamed tissues along fractalkine or IL-8 in view of their higher expression of CX3CR1^+^ and CXCR1^+^ and lower proportion of CD62L.

Analysis of transcription factors of these novel CD34^+^ cells showed that, they have a different array of transcription factors, including Tbet and FoxP3 in addition to Id2, E4BP4, which are expressed in classical CD34^+^DNAM^−^CXCR4^−^ cells purified from CB. A wide difference in transcriptional signature was further confirmed and expanded by microarray analysis of purified CD34^+^ cells. Interestingly, their abundant transcription of metalloproteases supports the idea of a direct exit in areas of osteoclast resorption. Further, and in line with previous reports that failed to pinpoint the exact nature of the progenitor cells (29, 67), this novel CLP could give rise to NK and T cells but not to myelomonocytes. On the contrary, CB-derived CD34^+^ cells give rise *in vitro* only to NK cells and myelomonocytes but not to T cells or NKT cells.

Interestingly, when considering the characteristics of NK cells *in vitro* derived from these precursors, some remarkable differences are evident in comparison to NK cells derived from CD34^+^DNAM^−^CXCR4^−^ progenitors. Lin^−^CD34^+^DNAM-1^bright^CXCR4^+^ derived maturing NK cells appear to have a much more mature and licensed phenotype, as they express KIRs and perforin, high levels of NCRs, DNAM-1 and NKG2D and also produce IFNγ when triggered. These characteristics are unseen in maturing NK cells derived from CD34^+^ CB cells, which under the same culture conditions do not produce IFNγ, and are NCR low, KIR^−^, DNAM-1^+/−^, and NKG2D^low/neg^.

The question remains open on where Lin^−^CD34^+^DNAM-1^bright^CXCR4^+^ cells fit with the classical known human NK cell progenitor hierarchy (18, 19) and why only very low levels of circulating CLP are detectable in HDs (16), while they may be greatly increased during systemic inflammation. According to the study by Doulatov et al. (19), Lin^−^CD34^+^DNAM-1^bright^CXCR4^+^ cells would (surprisingly) fit in the group of megakaryocyte/erythroid precursors, characterized by the CD38^+^CD10^−^CD7^−^Flt3 phenotype similar to that of Lin^−^CD34^+^DNAM-1^bright^ cells. In the absence of experiments carried out with culture conditions favoring different pathways for precursor differentiation, one cannot exclude that different progenies might be obtained.

**Figure 1:**
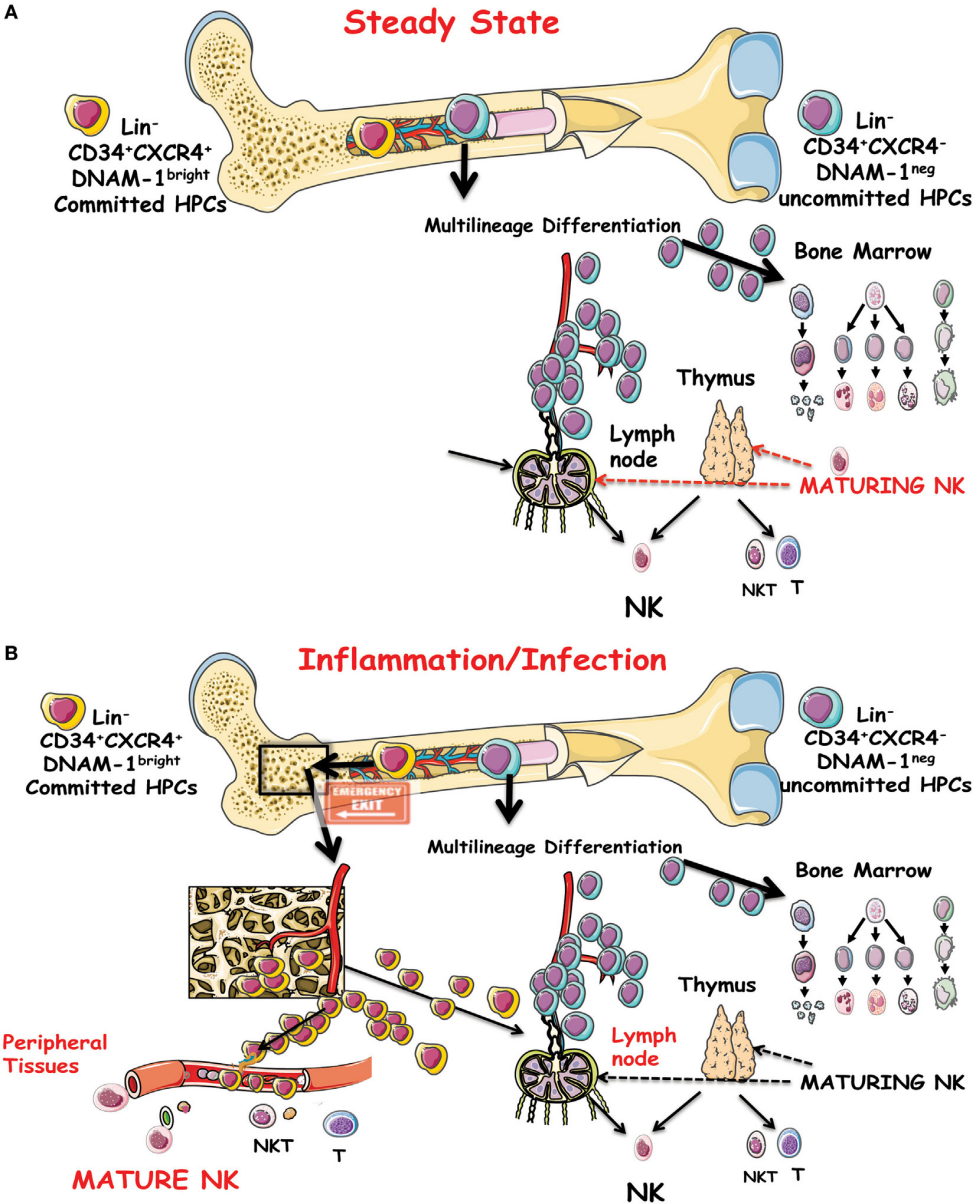
**(A)** Schematic representation of the precursor genesis and maturation process of NK cells *in vivo* under steady-state conditions. CD34^+^DNAM-1^−^CXCR4^−^ cells are indicated as general precursors to subsequent stages of multilineage commitment. A committed lymphocyte precursor to NK cells in the bone marrow (BM) or entering the thymus or lymph nodes gives rise to canonical maturing NK cells. **(B)** Under inflammatory conditions, a CD34^+^DNAM-1^bright^CXCR4^+^ cell precursor presumably resident in a BM niche proximal to osteoclasts is released into the bloodstream. These cells may travel to peripheral tissues in addition to secondary lymphoid organs and generate differently mature NK cells in addition to T and NKT cells.

Lin^−^CD34^+^DNAM-1^bright^CXCR4^+^ NK cell progeny includes a full array of the classical phenotypes, including CD56^bright^, CD56^dull^, and CD56^−^CD16^+^ NK cells subsets in addition to NKT CD3^+^CD56^+^ cells and to T cells but no cells of monocyte/myelomonocytic lineage (68). In view of the quite different phenotype of maturing NK cells derived from these precursors *in vitro* as compared to NK cells maturing from CD34^+^DNAM-1^−^CXCR4^−^ CB cells, it is tempting to hypothesize that the so far acknowledged model for NK cell differentiation and maturation from a single progenitor into all the known phenotypes and subsets may need renewed evaluation (Figures 1A,B).

Since an until recently uncharacterized CD34 precursor with distinct transcriptional signature and phenotype gives rise to NK cells with different phenotypic and functional characteristics, the hypothesis may be proposed that a parallel development of some NK cell phenotypes may take place *in vivo* from two different CD34^+^ precursors (i.e., Lin^−^CD34^+^DNAM-1^bright^CXCR4^+^ and CD34^+^DNAM-1^−^CXCR4^−^). It is, therefore, possible that a good number of the diverse NK cell phenotypes observed by mass cytometry (12) and possibly also some of the special subsets of NK cells observed *in vivo* may derive from different developmental stages of the two CD34 precursors. This view could be in line with the data by Doulatov et al. and Laurenti et al. (18, 19) where different CLP may give rise to different progenies but the same progeny may derive from different CLPs (17, 18). In addition, it should be underscored that recent work by Wu and colleagues (69) in elegant experiments of clonal tracking has identified a quite surprising origin of NK cells in macaques. Barcoding experiments show that in these animals, a progenitor different from B/T/Myeloid lineage stem cells gives rise to CD16^+^CD56^−^ NK cell progeny, and more importantly, parallel development of different NK cell phenotypes derive from different progenitors. Given the difficulties in defining CD56^bright^ and CD56^dim^ NK cell subsets in chimpanzees and macaques (70), the possible correlate of these findings on clonal tracking in macaques (69) needs to be evaluated with caution and may deserve evaluation also in other non-human primates. Overall, the identification of a novel CD34^+^ cell, giving rise to NK cells with distinct characteristics may represent a parallel and concurrent reading frame for the established model of NK cell development from CD34^+^ cells to CD56^bright^ to CD56^dim^ NK cells (9, 10).

## Concluding Remarks

The identification of a novel CD34^+^ cell with distinct transcriptional and phenotypic characteristics to standard CD34^+^ cells, endowed with the ability to generate NK cells of a special phenotype, opens new possibilities to improve our understanding of NK cell development and maturation, particularly under special or emergency conditions including systemic inflammation.

In this regard, several points need to be further understood to formulate a clear picture.

First, it will be important to understand whether additional different CD34^+^ precursors exist that may give rise preferentially to specific NK cell (or ILC) progenies or whether the identification of Lin^−^CD34^+^DNAM-1^bright^CXCR4^+^ cells represents the only other CD34^+^ precursor, giving rise to NK cells. In other terms, we do not yet know whether these cells were the only ones that needed characterization in a sort of “dark side of the (BM) niche” that eluded identification until recently.

Next, it will be important to understand the relationship of these NK cells that are actively released from osteoclast niches to classical CD34^+^DNAM-1^−^ NK cells that are passively released in the sinusoids.

Finally, careful analysis of NK cells developing from Lin^−^CD34^+^DNAM-1^bright^CXCR4^+^ cells is needed to understand whether these presumably “tissue-bound” precursors follow a predefined program or rather whether signals delivered in peripheral tissues guide and sustain the development of NK cells with specialized function.

## Author Contributions

FB analyzed, discussed, and interpreted data and literature and wrote the manuscript; FM analyzed, discussed, and interpreted data and literature and contributed to manuscript preparation; AM supervised, discussed, and interpreted data and literature, verified data analysis, and wrote the manuscript.

## Conflict of Interest Statement

The authors declare that the research was conducted in the absence of any commercial or financial relationships that could be construed as a potential conflict of interest.
